# Efficient Size-Dependent Hot Electron Transfer from Au to TiO_2_ Nanoparticles

**DOI:** 10.1021/acs.nanolett.4c06154

**Published:** 2025-02-14

**Authors:** Nandan Ghorai, Zhicheng Yang, Sara T. Gebre, Shengxiang Wu, Fengyi Zhao, Ilia N. Ivanov, Tianquan Lian

**Affiliations:** ¶ Department of Chemistry, Emory University, 1515 Dickey Drive, Atlanta, Georgia 30322, United States; ¥ Center for Nanophase Materials Sciences, Oak Ridge National Laboratory, Oak Ridge, Tennessee 37831, United States

**Keywords:** Au nanoparticles, TiO_2_, surface plasmon resonance, hot electron transfer, plasmon damping

## Abstract

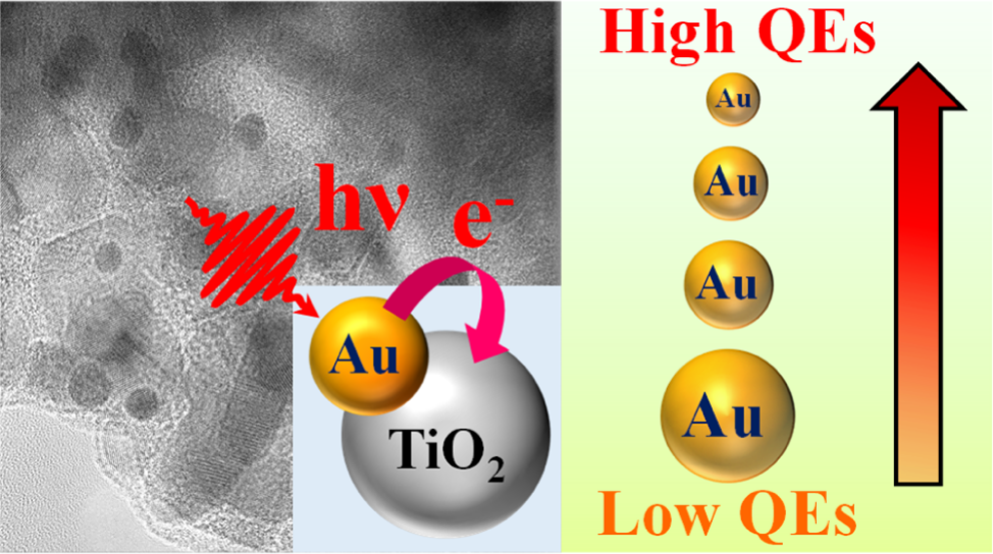

Harvesting of plasmon-induced hot carriers at the metal/semiconductor interface offers a promising and innovative avenue for solar energy conversion. However, their practical implementation is often hampered by their limited efficiencies. Herein, we have demonstrated a highly efficient plasmonic hot electron transfer with a quantum efficiency (QE) of up to 57 ± 4% from 5.25 nm Au nanoparticles (NPs) to TiO_2_ films under 400 nm ultrafast laser excitation. The observed hot electron transfer QEs decrease at larger particle sizes, to 20% for 9.1 nm Au, and show negligible changes with excitation wavelengths at 400, 500, and 600 nm. Analysis of the size and excitation wavelength dependent hot electron transfer QEs suggests they contain contributions of interband absorption, indirect plasmon-induced hot electron transfer (PHET), and direct plasmon-induced interfacial charge transfer transition (PICTT) pathways, and QEs of all three pathways increase at smaller Au size. Our result suggests that reducing plasmon particle sizes is a promising approach for efficient plasmonic hot-carrier extraction.

Plasmonic metallic nanomaterials integrated within a semiconductor matrix have found applications in photocatalysis, photovoltaics, and photodetectors because they enhance the light harvesting capabilities of the semiconductor and/or enable hot-carrier transfer across the metal/semiconductor interface.^1−7^ Hot electron transfer at the Schottky junction at the metal/semiconductor interface is often thought to occur through the conventional indirect plasmon-induced hot electron transfer (PHET) pathway.^8−12^ The efficiency of the PHET process depends on two sequential steps: hot electron generation (HEG) in the metal nanoparticles (NPs) and hot electron transfer (HET) across the interface. In metals such as Au, plasmons can decay through the interband excitation of electrons from the d-band to sp-band and or the intraband (sp → sp band) excitation of electrons from below to above the Fermi level, and the interband pathway dominates in large Au particles, leading to a low HEG quantum efficiency (QE) of hot electrons above the Schottky barrier.^13−15^ Furthermore, HET competes with hot electron relaxation in metal to lower the HET QE, which further lowers the overall PHET QE.^13−15^

In recent years, significant research effort has been devoted to developing approaches to improve the plasmonic hot electron transfer QE, and two effective approaches have been reported.^16−18^ In the first approach, by controlling the plasmonic particle size, the relative contributions of intraband and interband plasmon decay pathways can be affected to alter the hot electron energy distribution. For example, it was shown that the PHET QE in Au-tipped CdS nanorods can be enhanced from 1% to 18% by reducing the Au NP size from 5.5 to 1.6 nm.^18^ In the second approach, strong coupling between the metal and semiconductors can lead to chemical interface damping (CID), in which plasmons can decay by directly exciting an electron across the metal/semiconductor interface through plasmon-induced interfacial charge transfer transition (PICTT).^10,19−23^ For example, it was demonstrated that hot electron transfer in Au-tipped CdSe nanorods is dominated by the PICTT pathway with an impressive QE of ∼24%.^18^ It is further demonstrated that in Ag/TiO_2_ films both the PHET and PICTT contribute to the plasmonic hot electron transfer and QEs of both processes increase at small Ag particle sizes, leading to a total hot electron transfer QE of up to 53% in small Ag NPs.^16^

In this work, we aim to examine whether the previously reported approaches for improving plasmon-induced hot electron transfer can be extended to the Au/TiO_2_ interface by systematically examining the hot electron transfer QE as a function of the Au particle size. Although plasmon-induced hot electron transfer at the Au/TiO_2_ interface has been reported,^7,24−26^ there have not been systematic examinations of its size dependence and the HET mechanism remains unclear. Furthermore, hot electrons can also be generated by interband absorption within a metal, and it remains unclear whether interband excitation can also lead to efficient hot electron transfer at the metal/oxide interface. Using transient IR absorption spectroscopy and by using Ru(dcbpy)_2_(NCS)_2_ [dcbpy = bis(4,4′-dicarboxy-2,2′-bipyridine)]-sensitized anatase nanoporous TiO_2_ films (RuN3/TiO_2_) as a reference of 100% electron transfer, we measured the hot electron transfer at the Au/TiO_2_ interface as a function of Au particle size. We observe that the HET QE increases at smaller Au particles similar to the previous report of Ag/TiO_2_ and an impressively high HET QE of 57 ± 4% can be achieved at 400 nm excitation for small Au NPs (∼5.25 nm). Analysis of the size and excitation wavelength dependence of plasmon damping and HET QE enables us to identify the relative contributions of interband excitation, PHET and PICTT pathways, and their dependence on the Au particle size.

Au NPs of different sizes were synthesized following previous literature procedures with minor modifications.^27^ Four colloidal Au particle samples with average diameters of 7.86 ± 0.65, 4.56 ± 0.29, 4.10 ± 1.00, and 3.56 ± 1.29 nm are used in this study. Au/TiO_2_ thin films were fabricated by following the previous procedures provided in the Supporting Information (SI). This resulted in four Au/TiO_2_ samples with average Au diameters of 9.13 ± 1.43, 8.51 ± 1.49, 6.86 ± 1.03, and 5.25 ± 0.89 nm. These colloidal Au particles and Au/TiO_2_ films are referred to as Au_x.x_ and Au_x.x_/TiO_2_, respectively, with x.x being the first two digits of the average Au particle size. A representative transmission electron microscopy (TEM) image of colloidal Au_4.5_ NPs is shown in Figure 1a, alongside an inset showing a magnified TEM image and a distribution histogram of the particle size. Figure 1b represents high-resolution TEM of Au_8.5_/TiO_2_ films, revealing interplanar spacings of 0.21 and 0.35 nm corresponding to the (200) and (101) lattice planes of Au and TiO_2_, respectively.^28,29^ A complete set of TEM images and size distributions of colloidal Au and Au NPs in TiO_2_ films are shown in Figure S2 and Figure S3, respectively.

**Figure 1:**
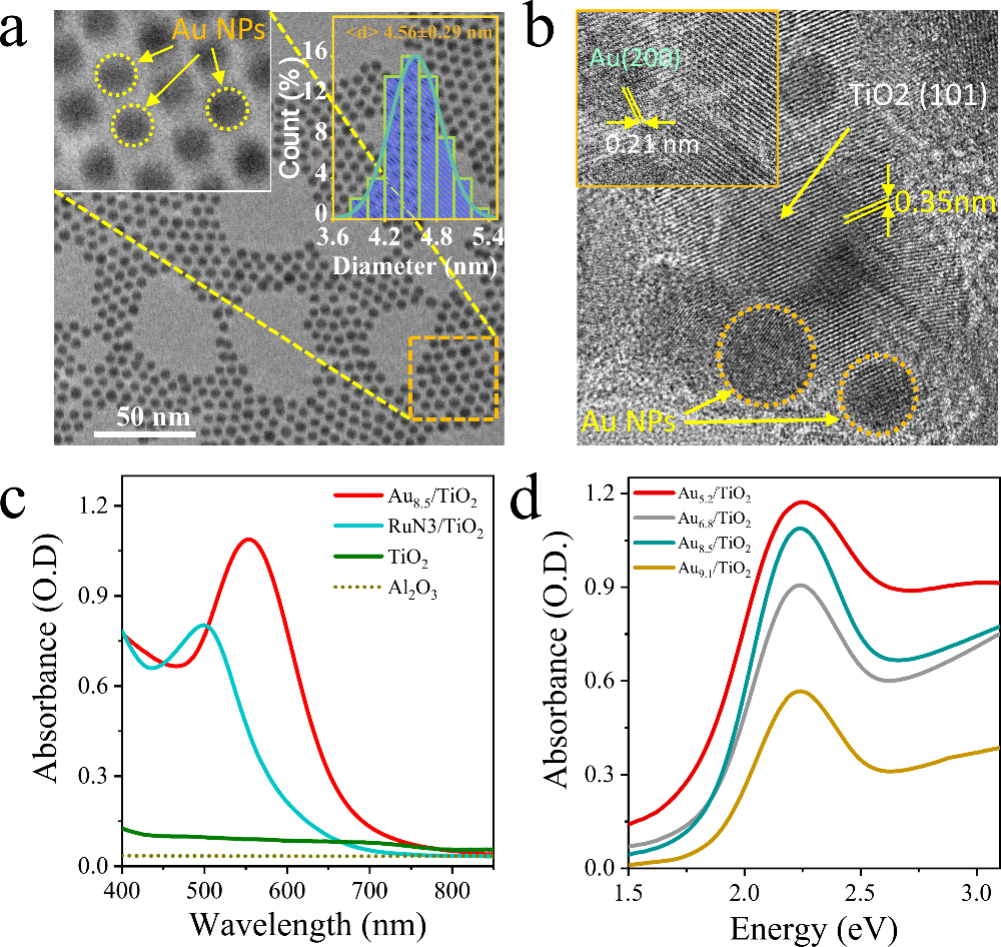
(a) Transmission electron microscopy images of Au_4.5_ NPs. Inset: magnified image of the selected area (left) and size distribution histogram of the synthesized gold NPs (right). (b) High-resolution TEM image of Au_8.5_/TiO_2_ films. The interplanar spacing of the anatase TiO_2_ film is approximately 0.35 nm. (c) Steady state absorption spectra of Au_8.5_/TiO_2_, RuN3/TiO_2_, and TiO_2_ and a bare sapphire window. (d) Comparison of UV–vis absorption spectra calculated from DRS of all fabricated Au/TiO_2_ films.

The steady-state UV–vis absorption spectra of Au_8.5_/TiO_2_, RuN3/TiO_2_, TiO_2_, and Al_2_O_3_ are shown in Figure 1c. Figure 1d and Figure S4a show the size-dependent extinction spectra of Au particles in solution, respectively. In this size regime, the scattering due to Au NPs is negligible and the extinction spectra of Au NPs in solutions can be considered as their absorption spectra. For Au/TiO_2_, there is considerable scattering due to the nanoporous TiO_2_ film. The absorption spectra of Au/TiO_2_ are calculated from the measured transmission spectra and diffuse reflection spectra (DRS), as shown in Figure S5a,b,c,d and described in Figure SI4. As shown in Figure S4b,c, compared to colloidal Au particles in solution, the surface plasmon resonance (SPR) band of Au NPs on TiO_2_ films shows a broadened width and red-shifted peak position. To obtain the plasmon bandwidth, steady state absorption spectra were fitted with the sum of the plasmon band and interband absorption of Au (Figure 2a, Figure S6, Figure S7, and Table S2) following the procedure described in SI5.^16,27^ The fit also reveals how the total absorbance at each wavelength (*OD*_*tot*_) can be decomposed into a sum of plasmon absorption (*OD*_*pla*_) and interband absorbance (*OD*_*int*_). The percentages of absorbed photons by plasmon absorption, i.e., *OD*_*pla*_/*OD*_*tot*_, are 78%, 49%, and 7% at 600, 500, and 400 nm, respectively. The SPR width as a function of 1/*R* for both Au colloidal particles and Au/TiO_2_ films is shown in Figure 2b, and the total plasmon damping rate is fit to the sum of bulk (*γ*_*bulk*_), surface (*γ*_*surf*_), and interfacial damping or CID (*γ*_*int*_) rate.^17,30,31^ The fit reveals that both *γ*_*surf*_ and *γ*_*int*_ increase linearly with 1/*R*. Detailed analysis is provided in SI6.

**Figure 2:**
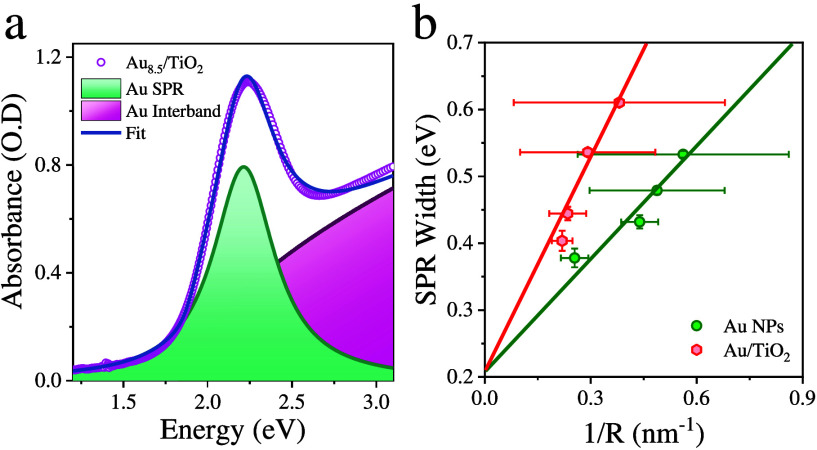
(a) Measured steady state absorption spectrum of Au_8.5_/TiO_2_ and its fit (blue-gray line) to the sum of three components: the Au SPR band (dark cyan area) and Au interband absorption (dark red area). (b) The SPR band widths of pure gold NPs (filled circles) and Au/TiO_2_ (filled diamonds) NPs as a function of 1/*R* (where *R* is the gold nanoparticle radius) and their linear fits (dashed line).

Ultrafast mid-infrared transient absorption spectroscopy was utilized to investigate the size-dependent hot electron transfer at the Au/TiO_2_ interface with 400, 500, and 600 nm excitation. All excitation wavelengths fall below the interband excitation threshold of anatase TiO_2_ (∼380 nm). Photoinduced electron transfer to the TiO_2_ conduction band (CB) introduces a broad intraband mid-IR absorption, as shown in Figure 3a. The ET kinetics of Au/TiO_2_ and RuN3/TiO_2_ films measured with an excitation wavelength of 500 nm and probe wavenumber of ∼2222 cm^–1^ are shown in Figure 3b, Figure S9a (400 nm), and Figure S9c (600 nm), respectively. In these figures, the TA signal amplitudes have been scaled to correspond to the same number of absorbed photons by both Au NPs and RuN3 dye molecules at the excitation wavelength, and the contribution of direct excitation of TiO_2_ has also been subtracted, following the procedure described in SI8. The rise time of the mid-IR TA signal is attributed to ET from RuN3 and/or Au NPs to TiO_2_.^7,16^ The decay of the TA signal corresponds to back electron transfer to RuN3 or Au from the CB of TiO_2_ and hot electron relaxation within TiO_2_.^16,32^ The transient mid-IR absorption kinetics can be fit by a model developed previously for RuN3/TiO_2_ and Ag/TiO_2_, as described in detail in SI8 (Figure S8).^16,32^ At 500 nm excitation, for Au_8.5_/TiO_2_, the rise component of N_e_(*t*) shows a time constant of 0.17 ± 0.03 ps, and its decay can be fitted by three exponential decay functions, with time constants (and amplitudes) of 2.40 ± 0.7 (33%), 17.6 ± 18 (36%), and ≫1 ns (30%), similar to those observed in Au/TiO_2_ and Ag/TiO_2_ films.^16,25^ From the fitting parameters, the half-life of population decay (due to back electron transfer) can be calculated as 6.6 ps. All fitting parameters are summarized in Tables S4, S5, and S6 for kinetics measured with excitation at 400, 500, and 600 nm, respectively. Analysis of the fitting parameters reveals that the rise time and recovery time for Au NPs of different sizes are similar, suggesting that both the ET time and the back ET time are independent of the size of the NPs. Furthermore, these kinetics are also similar at different excitation wavelengths.

**Figure 3:**
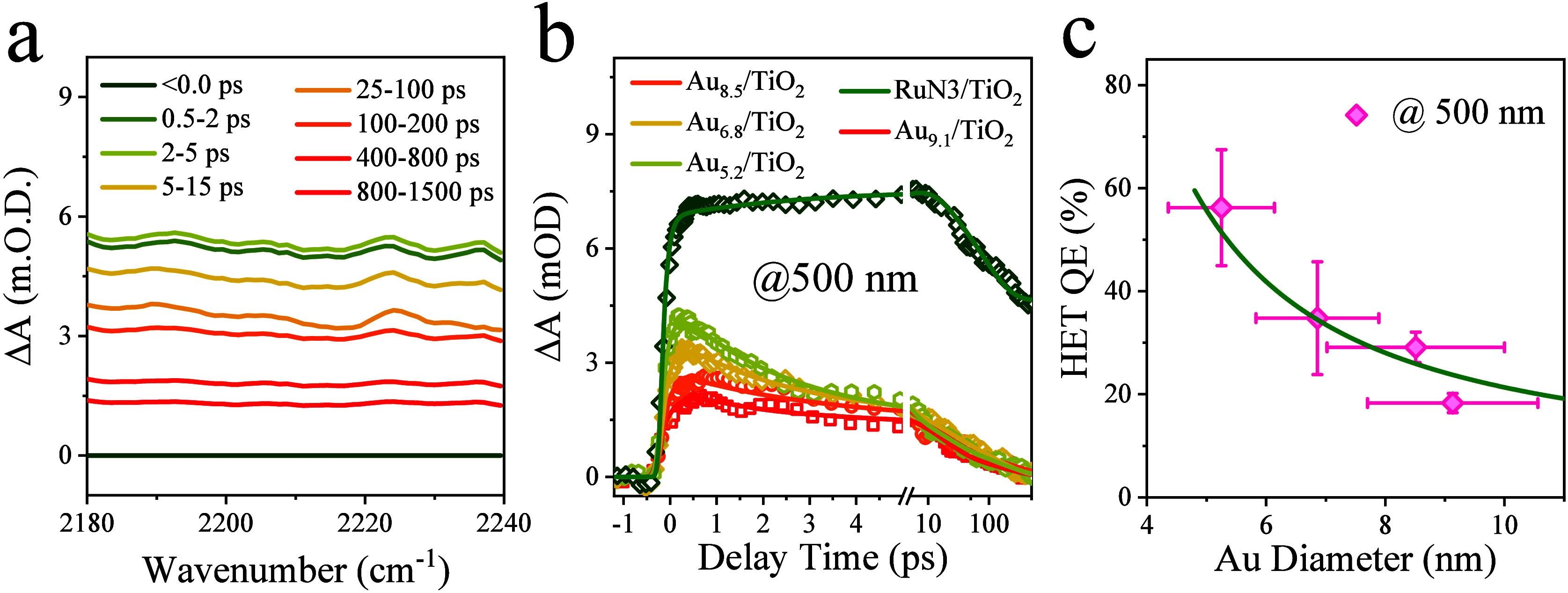
Transient IR spectra and kinetics of the Au/TiO_2_ film under ambient conditions measured with 500 nm excitation. (a) Transient absorption spectra of the Au_8.5_/TiO_2_ film at indicated delay times after photoexcitation of the Au particle, showing the intraband absorption of injected electrons in TiO_2_. (b) Size-dependent electron injection kinetics for Au/TiO_2_ films of different Au size and the RuN3/TiO_2_ film monitored at 2222 cm^–1^. (c) Hot electron transfer QE as a function of Au particle size. Excitation fluence: ∼0.61 W/cm^2^.

From the fitting results, we can determine the relative population of injected electrons in TiO_2_ for RuN3/TiO_2_ and Au/TiO_2_ samples of different Au sizes at different excitation wavelengths. It has been reported that the electron injection QE in RuN3/TiO_2_ is unity.^33^ Thus, by comparing the maximal injected electron populations in Au/TiO_2_ to that in RuN3/TiO_2_ and by accounting for the number of absorbed photons in these samples, we can determine the hot electron transfer QE in Au/TiO_2_ samples. As shown in Figure 3c, the HET QEs for Au/TiO_2_ at 500 nm excitation were found to decrease at larger Au particle size, changing from 56 ± 11% for the smallest (5.25 nm) Au particles to 18 ± 2% for the largest (9.1 nm) Au particles. Similarly, the size-dependent trends for the HET QEs are observed at 400 nm excitation (Figure S9a,b) and 600 nm excitation (Figure S9c,d). Pump fluence dependent electron injection electron kinetics traces are presented in Figures S10 and S11 and indicate no multiphoton absorption (Figure S12). TAIR of Au_8.5_/Al_2_O_3_ shows negligible signal, and bare TiO_2_ films show much smaller mid-IR signal (Figure S13). The latter represents the contribution of direct excitation of TiO_2_ and has been subtracted from the total signal of Au/TiO_2_, as described in SI8. The measured HET QEs as a function of Au NP size at different excitation wavelengths are compared in Figure 4a.

**Figure 4:**
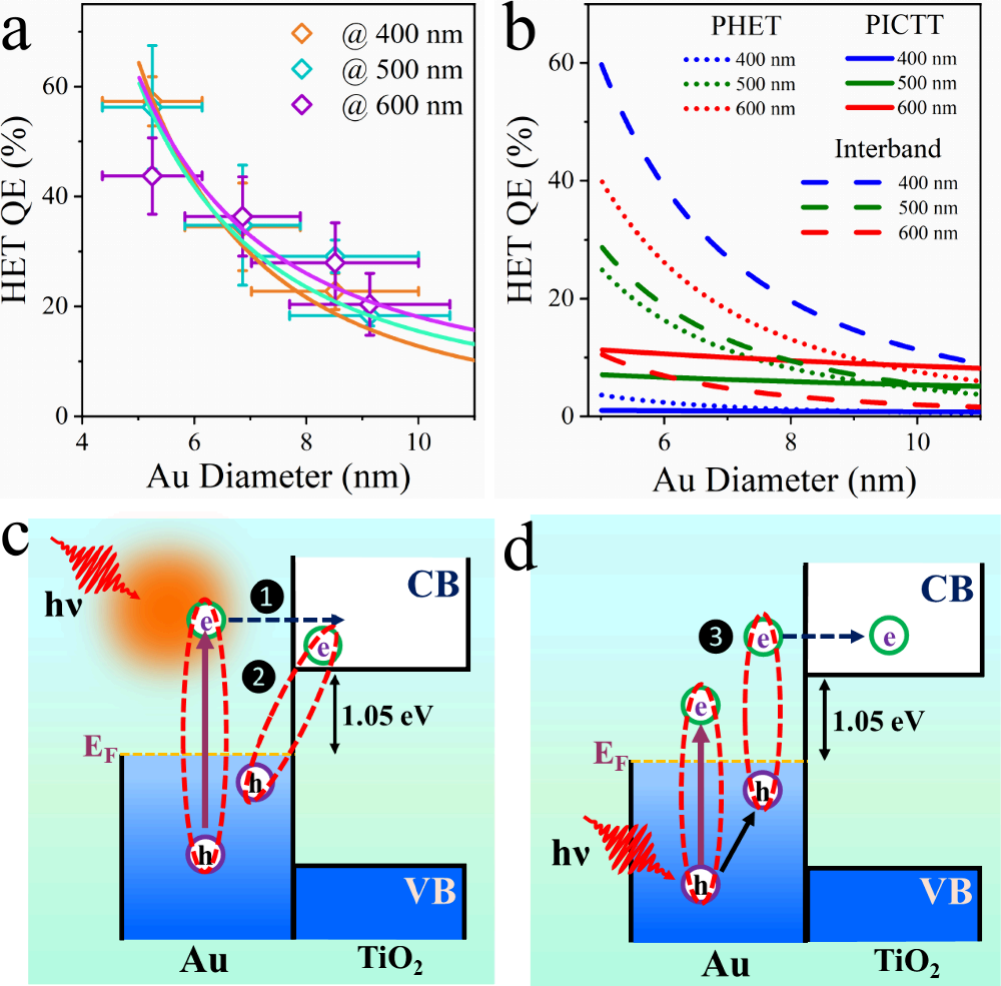
Size and excitation wavelength dependent HET in Au/TiO_2_ films. (a) Size dependence of HET efficiency after excitation at 400, 500, and 600 nm. (b) The calculated QE HET from Au to TiO_2_ via interband excitation (dashed line) and PHET (dotted lines) and PICTT (solid lines) pathways of plasmon excitation, as a function Au particle size at indicated excitation wavelengths (400, 500, and 600 nm). (c) Schematic representation of plasmon-driven HET processes via (1) PHET and (2) PICTT pathways. (d) Schematic representation of hot electron transfer resulting from interband excitation in Au/TiO_2_ films.

The total hot electron transfer quantum efficiency is given by eq 1:
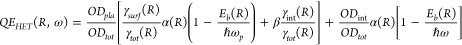
1

In eq 1, the total hot electron transfer QE consists of the contributions of plasmon excitation and interband excitation, with the percentages of absorbed photons of , and , respectively. Following our previous model,^16^ plasmon excitation can proceed by indirect PHET and direct PICTT pathways. Surface damping gives rise to hot ET through the PHET pathway, while interfacial damping leads to hot electron transfer through the PICTT pathway (Figure 4c). The sequential PHET, process (1) in Figure 4c, is given by the first term in eq 1.^16^ The decay of plasmons within Au is primarily through the generation of electron–hole (e-h) pairs via sp–sp intraband and d–sp interband transitions.^14,34^ Because the Au d-band is ∼1.8 eV below the Fermi level, the interband plasmon decay pathway deposits most of the plasmon energy in the hole in the d-band, with negligible hot electrons at energy above the TiO_2_ CB edge. In small plasmonic particles, enhanced surface damping facilitates plasmon decay in intraband excitation, which generates a nearly uniform distribution of hot electrons from the Fermi level to the plasmon energy. Thus, the probability of generating hot electrons (QE_HEG_) is assumed to be proportional to the ratio of surface to total damping (), which is the probability of plasmon decay by surface damping.^35^ The probability of transferring the hot electrons into TiO_2_ is assumed to follow the modified Fowler model, ,^16,36^ in which the size-dependent barrier height, i.e., the energy difference between the TiO_2_ CB edge and Au Fermi level, is given by *E*_b_(*R*) = *E*_b0_ – ,^37,38^ with *E*_b0_ = 1.05 eV,^35^ ε ≈ 28 for anatase,^35,39^ and α(*R*) is a scaling factor to account for the competition of hot electron transfer and cooling. Here, we have assumed that the plasmon band is dominated by homogeneous broadening and regardless of the excitation wavelength, and the same plasmon band is excited at 400, 500, and 600 nm, although the relative contribution of interband vs plasmon excitation varies at these wavelengths. The contribution of the PICTT pathway, process (2) in Figure 4c, is given by the second term in eq 1. Here we assume that the probability of plasmon decay by PICTT is given by the quotient of the interfacial damping and the overall plasmon damping rate, , and β is the scaling factor that accounts for the probability of PICTT through the Au to TiO_2_ CB transition.^21^

The third term in eq 1 represents the contribution of hot electron transfer through interband excitation (Figure 4d). In our previous study of hot electron transfer, we have assumed that plasmon excitation dominates the absorption at the excitation wavelength.^16−18^ This assumption is not valid in Au/TiO_2_ at 400 nm excitation in which the absorption is dominated by the Au interband transition. We assume that the transfer of hot electrons generated by interband absorption also follows the modified Fowler model as the PHET pathway. Interband absorption deposits most of the energy in the d band, which should not lead to efficient transfer of the hot electron to TiO_2_. However, the hot holes in the d band can exchange energy rapidly with the electron through scattering processes to generate energetic electrons above the barrier *E*_b_, as illustrated in Figure 4d.^26,40^

We have fitted the experimentally measured HET QEs at 400, 500, and 600 nm excitation according to eq 1, as shown in Figure 4a. The model captures the size-dependent QEs observed in Au/TiO_2_ films at all excitation wavelengths. The contributions of the interband absorption and PHET and PICTT pathways are shown in Figure 4b. Best fit of the data yields β = 0.44, which indicates that about half of the PICTT pathways involves the Au to TiO_2_ CB interfacial charge transfer transition, and the other interfacial charge transfer transitions, i.e., the transition from the TiO_2_ valence band to unfilled orbitals in Au sp bands, contributes to the remaining half of the decay. Interestingly, this value is similar to the previously reported β value of 0.41 in Ag/TiO_2_ samples,^16^ although it is unclear what determines the exact partition of different PICTT pathways. The PICTT contribution increases at smaller Au particles, which within our model is determined by the increase of interfacial damping at small particle size. The best fit also reveals α(*R*) = 17.2/(*R*/nm)^2^, which increases from a value of ∼0.5 to 2.5 when the Au particle size decrease from 9.1 to 5.3 nm, accounting for the increase of PHET QE at smaller Au particles. This trend reflects a more effective interfacial ET compared to hot electron cooling at smaller Au sizes. The interband contribution is modeled in the same way as the PHET and also exhibits similar increases at smaller particle sizes.

At different excitation wavelengths, the relative contribution of interband versus plasmon decay changes. According to the fit to the absorption spectra, at 400 nm, interband excitation accounts for 93% of the absorbed photons, while at 600 nm, 78% of the absorbed photons leads to plasmon excitation. Interestingly, as shown in Figure 4a, the observed hot electron transfer QEs vary negligibly with the excitation wavelength for most particle sizes, except for the smallest Au particles. We attribute this to efficient HET transfer through the interband absorption process, which has an efficiency similar to that of plasmon excitation. Within our model, the surprisingly efficient interband HET pathway implies that the hot d band holes generated by interband absorption can efficiently transfer energy to generate hot electrons in the sp band through scattering processes. Without assuming this fast energy transfer from hot holes to electrons, our model will not be able to explain the high hot electron QE at 400 nm excitation.

Previously, Furube et. al reported an electron injection QE of ∼40% from ∼10 nm Au to TiO_2_ at 500 nm excitation.^7^ Ratchford et al. reported for ∼10 nm Au on TiO_2_ the hot electron transfer QE increase from 25% at 750 nm excitation to 45% at 550 nm excitation.^25^ Our results are consistent with these previous reports of plasmonic HET in similar Au/TiO_2_ heterojunctions. More recently, Link and co-workers reported an electron transfer QE of ∼45% in Au/TiO_2_ core–shell nanorods (26 nm × 55 nm Au nanorods).^26^ Furthermore, according to our model, the hot electron transfer QE depends on the contribution of the CID, which is sensitive to the nature of the Au/TiO_2_ interface. Inspection of absorption spectra of these samples suggests different degrees of Au plasmon band broadening in these samples, which may be an indication of their varying rates of CID and may account for the different electron transfer QEs in these reports. All these HET QEs are much higher than those observed in Au-tipped CdS nanorods, in which only the PHET pathway contributes.^18,41^ The observed size-dependent HET QE is similar to our previous report of HET of Ag/TiO_2_, which also shows contributions of PHET and PICTT pathways,^16^ although considerably smaller Ag particles (<6 nm) were used. In a previous study of Au/TiO_2_, the charge recombination time was found to be dependent on the Au particle size. This is not observed in our work, and the reason for this difference is not clear.^42^ These results suggest the importance of both the metal and semiconductor materials and their interfaces in affecting the HET QE.

In summary, with decreasing Au NP particle size in Au/TiO_2_, both the surface and interfacial damping rate of the plasmon band increases, and the HET QEs increase from 18% for 9.1 nm Au NPs to 56% for 5.2 nm Au NPs at 500 nm excitation. For Au_8.5_/TiO_2_ at 500 nm excitation, the electron transfer time is 0.17 ps and the back electron transfer process can be well fit by three exponential decays with a half-life of 6.6 ps. The hot electron transfer and decay kinetics are similar for Au particles of different size and at different excitation wavelengths. The size and excitation wavelength dependent HET QEs can be fit by a model that accounts for the contributions of interband absorption, indirect plasmon-driven PHET, and direct plasmon-driven PICTT pathways. According to this model, the HET QEs of the PHET pathway show a much larger size dependence compared to PICTT, and the QEs of interband absorption show a similar size dependence to the PHET pathway. For example, at 500 nm excitation, when the Au particle size decreases from 9.1 to 5.2 nm, the PHET QEs increase from ∼6.0% to 22.3%, PICTT QEs increase from ∼5.6% to ∼6.9%, while interband HET QEs increase from ∼6.8% to 25.6%. At 600 nm excitation, the contribution of plasmon decay dominates, and at 400 nm interband absorption dominates. However, because the interband and PHET pathways are similarly efficient, the observed HET QEs show similar QEs and Au size dependence at different excitation wavelength. The surprisingly efficient HET through interband absorption suggests that the hot holes in the d band generated by interband absorption can efficiently transfer its energy to the sp band electron to enable efficient HET. Our findings indicate that reducing plasmonic particle size effectively enhances both interband excitation and plasmon-induced hot electron transfer efficiency at metal/semiconductor interfaces.

## Supporting Information Available

Materials and methods, transmission electron microscopy, absorption and extinction spectra of Au NPs and Au/TiO_2_ films, fitting parameters of absorption spectra of colloidal Au NPs and Au/TiO_2_ films, mid-IR transient absorption setup, background correction and QEs of the hot electron transfer in a scattering sample, plasmon damping fitting, electron injection kinetics of Au/TiO_2_ and RuN3/TiO_2_ films, pump power dependent TA kinetics of Au/TiO_2_ and RuN3/TiO_2_ films, Fowler model for PHET QE calculation, pump power dependent TA kinetics of Au/TiO_2_ and RuN3/TiO_2_ films, control experiment and electron injection signal of Au/Al_2_O_3_ and bare TiO_2_ films (PDF)
