# Supplementary material for: Efficient Size-Dependent Hot Electron Transfer from Au to TiO2 Nanoparticles
Source: Nano Lett. 2025 Feb 14;25(8):3253–8. doi: 10.1021/acs.nanolett.4c06154 (PMC11869363; doi:10.1021/acs.nanolett.4c06154)
Supplement: Supplementary file 1 — nl4c06154_si_001.pdf [file nl4c06154_si_001.pdf]

Supporting Information

Efficient Size Dependent Hot Electron Transfer from Au to TiO_2 Nanoparticles

Nandan Ghorai[¶], Zhicheng Yang[¶], Sara T. Gebre[¶], Shengxiang Wu[¶], Fengyi Zhao[¶], Ilia N. Ivanov[¥], Tianquan Lian[¶*]

[¶]*Department of Chemistry, Emory University, 1515 Dickey Drive, Atlanta, GA 30322, USA*

[¥]*Center for Nanophase Materials Sciences, Oak Ridge National Laboratory, Oak Ridge, Tennessee 37831, USA*

Corresponding Author: Tianquan Lian

Email: tlian@emory.edu

Table of Contents

SI1. Materials and Methods

SI2. Transmission Electron Microscopy (TEM)

SI3. Extinction Spectra of Au NPs and Au/TiO_2 Films

SI4: Calculation of True Absorbance

SI5. Fitting Parameters of Absorption Spectra of Colloidal Au NPs and Au/TiO_2 Films

SI6: Plasmon Damping Fitting

SI7. Mid-IR Transient Absorption Setup

SI8: Background correction and QEs of the hot electron transfer in a scattering sample

SI9. Electron Injection Kinetics of Au/TiO_2 and RuN_3/TiO_2 Films

SI10. Fowler Model for PHET QE Calculation

SI11. Pump Power Dependent TA Kinetics of Au/TiO_2 and RuN_3/TiO_2 Films

SI12. Control Experiment and Electron Injection Signal of Au/Al_2O_3 and Bare TiO_2 Films

SI1. Materials and Methods

Materials: Hydrogen tetrachloroaurate (III) hydrate ($HAuCl_4 \cdot 3H_2O$), borane tert-butylamine borane complex (TBAB, 97%), 1,2,3,4-tetrahydronaphthalene (tetralin, anhydrous, 99%), oleylamine (OAm, ≥98% primary amine), oleic acid (OA, technical grad, 90%), and hexanes (for HPLC, ≥95%) were purchased from Sigma-Aldrich and used without further purification. Titanium dioxide (TiO_2) paste (18NR-T Transparent Titania Paste) was purchased from GreatCell Solar. The sapphire optical windows (25 mm in diameter, 2 mm in thickness) were purchased from Swiss Jewel Company. The RuN3 dye (cis-diisothiocyanato-bis (2,2'-bipyridyl-4,4'-dicarboxylic acid) ruthenium(II)) was purchased from Solaronix.

Synthesis of Small Gold (Au) Nanoparticles (NPs): We have synthesized small gold NPs after following the previous literature with some modifications.[1] Different sized Au NPs were synthesized by changing the reaction temperature. In the present study, we have chosen four reaction temperatures: 0 ^{0}C, 20 ^{0}C, 40 ^{0}C, and 60 ^{0}C. Briefly, an orange solution of tetralin (10 mL), OLM (10 mL) and $HAuCl_4 \cdot 3H_2O$ (0.1g) was heated at a specific temperature under argon (Ar). In a separate glass container, the reducing agent tetralin (1 mL), OLM (1 mL) and TBAB (0.5 mmol) were mixed by ultrasonication and injected into the aforementioned precursor solution. After the injection, the solution color changed to a deep purple color from orange and the mixture was kept for 1 hour before being cooled down to room temperature. The synthesized Au NPs of various sizes were precipitated with the addition of acetone and methanol and collected by centrifugation at 8000 rpm for 5 minutes.

TiO_2 Nanoporous Film: Detailed procedures are documented in a previous publication.[2] In brief, the purchased sapphire windows underwent a rigorous cleaning process. They were first subjected to a 24-hour immersion in aqua regia, followed by sequential rinsing with Milli-Q water and sonication in acetone and ethanol. Subsequently, the cleaned windows were dried using a stream of nitrogen gas.

The deposition of the TiO_2 nanoporous film was achieved by the doctor blade method onto the sapphire substrate. The film was then subjected to sintering at 500 °C for one hour to obtain the desired nanoporous structure.

Preparation of Au/TiO_2 Thin Film:

Deposition of Au NPs on the prepared TiO_2 nanoporous film: Au NPs were then deposited on the TiO_2 nanoporous film. This is achieved by soaking the TiO_2 film on the sapphire (Al_2O_3) window into a diluted Au NP solution in hexanes. The Au NPs were expected to adhere to the surface of the TiO_2 due to various interactions, such as van der Waals forces, electrostatic interactions, and chemical bonding.

Drying and removal of weakly absorbed Au NPs: After soaked the TiO_2 nanoporous film onto the Au NPs solution, it is left to dry in air. During the drying process, the Au NPs will adhere more strongly to the TiO_2 film. However, some Au NPs may only weakly bind to the surface or remain loosely bound. To remove these weakly absorbed Au NPs, the film is washed with hexane.

Heating at 500 °C to remove ligands: To create a clean and stable Au/TiO_2 film, the next step involves heating the prepared film at a high temperature of 500 °C for 1 hour. This elevated temperature effectively removes the ligands and other organic components from the Au NPs, leaving behind a well-defined Au/TiO_2 film.

Preparation of RuN3/TiO_2 Thin Film: The TiO_2 nanoporous film was immersed in a RuN3/ethanol solution for 1 hour to create a RuN3/TiO_2 composite (Figure S1). After immersion, the sample was washed with ethanol to remove weakly adsorbed RuN3 molecules, dried under N_2 flow, and stored in the dark before further experiments.

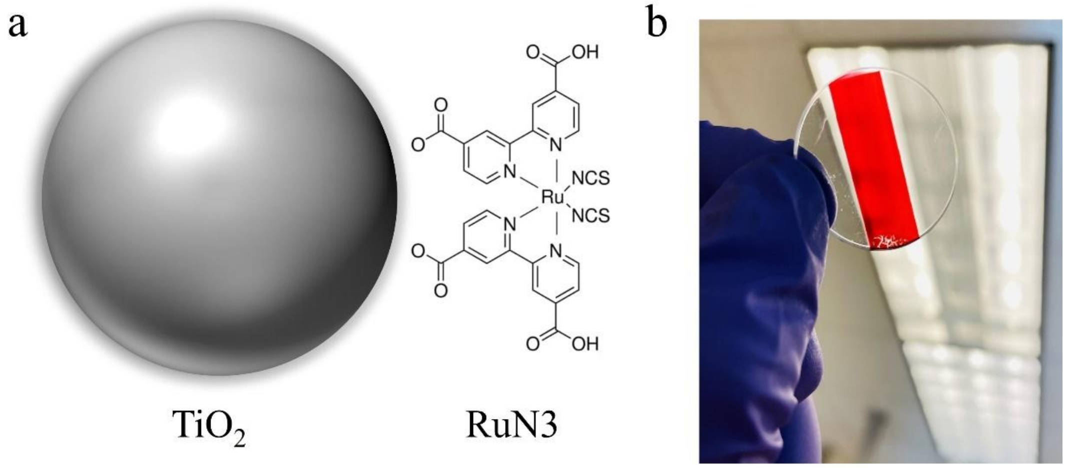

Figure S1: (a) Schematic illustration of RuN3/TiO_2 composite molecular structure. (b) Picture of Au/TiO_2 films.

SI2. Transmission Electron Microscopy (TEM)

TEM images were acquired using Hitachi 7700 and JEOL 1400 microscopes. Samples were drop casted on ultra-thin carbon coated Copper grids and imaged at 120 kV with aberration corrections. Colloidal Au NP sizes were 7.86 ± 0.62 nm, 4.56 ± 0.29 nm, 4.1 ± 1.00 nm, and 3.56 ± 1.29 nm, respectively. Figure S2 shows TEM images and particle size distributions of colloidal Au NPs after synthesis under different reaction temperatures.

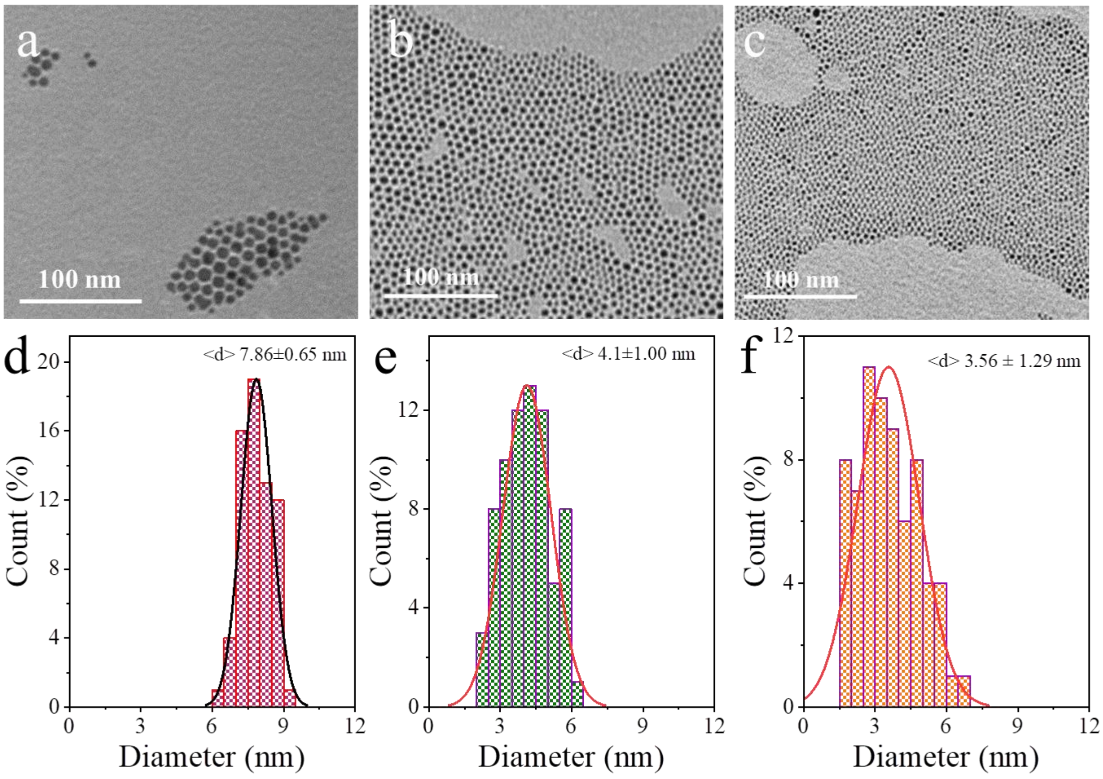

Figure S2: TEM images and size distribution histograms of colloidal Au NPs at reaction temperatures of (a, d) 0 ^{0}C, (b, e) 40 ^{0}C, and (c, f) 60 ^{0}C, respectively.

Similarly, we have demonstrated TEM images of Au/TiO_2 films and size distributions in Figure S3. Au NP size distributions in colloidal solution and films are summarized in Table S1. Size histograms show that Au NP sizes in films were 9.13 ± 1.43 nm, 8.51 ± 1.49 nm, 6.86 ± 1.03 nm, and 5.25 ± 0.89 nm, respectively. We observed small increases in NP size when it is embedded onto TiO2 surface.

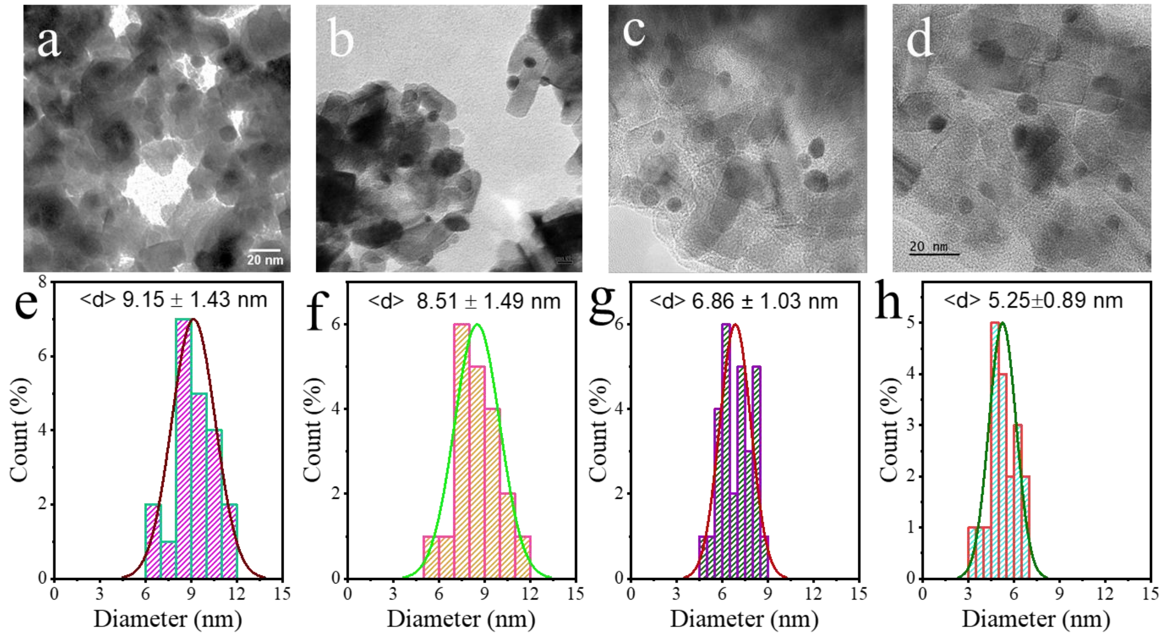

Figure S3: TEM images and size distribution histograms of Au NPs at reaction temperatures of (a, e) 0 ^{0}C, (b, f) 20 ^{0}C, (c, g) 40 ^{0}C, and (d, h) 60 ^{0}C in anatase TiO_2 films after breaking at 500 ^{0}C for 1 hour, respectively.

Table S1: Average diameters of the Au NPs in solution and films after heat treatment at 500 ^{0}C.

Reaction Temperature (^{0}C)	Average Au NPs size (nm)	Average Au size in film (nm)
0	7.86±0.65	9.13±1.43
20	4.56±0.29	8.51±1.49
40	4.10±1.00	6.86±1.03
60	3.56±1.29	5.25±0.89

SI3. Extinction Spectra of Au NPs and Au/TiO_2 Films

Steady state extinction measurements were carried out using an Agilent Cary 500 absorption spectrometer. Diffuse reflectance and transmitted spectra of Au/TiO_2 films were acquired by a Cary 5000 spectrometer equipped with integrating sphere. The spectrum (Figure S4) is accompanied by the absorption spectra of the Au/TiO_2 films and colloidal Au NPs for comparison. The presence of the TiO_2 film and the Au/TiO_2 film spectra provides insights into how the addition of TiO_2 affects the optical properties of the Au NPs. Figure S5 presents the measured transmission and diffuse reflectance spectra of Au/TiO_2 films with varying sizes of gold nanoparticles.

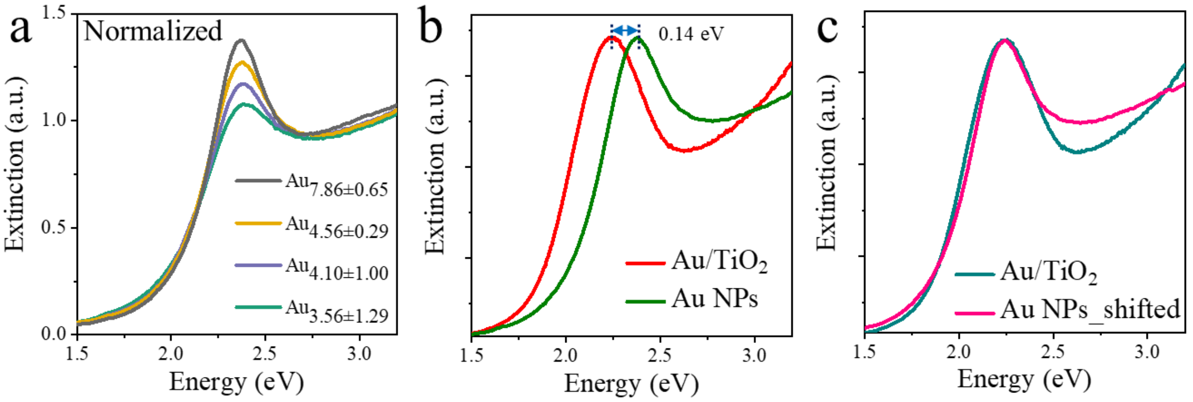

Figure S4: (a) Normalized steady state UV-Vis extinction spectra of colloidal Au NPs synthesized at different reaction temperatures. (b) Colloidal Au NPs (4.56 ± 0.29 nm) in hexanes and after depositing on TiO_2 films (baked at 500 ^{0}C). (c) Comparison of steady state absorption of $Au_{4.5}$ NPs and $Au_{8.5}/TiO_2$ film, showing that the plasmon band in Au/TiO_2 has been shifted by 0.14 eV.

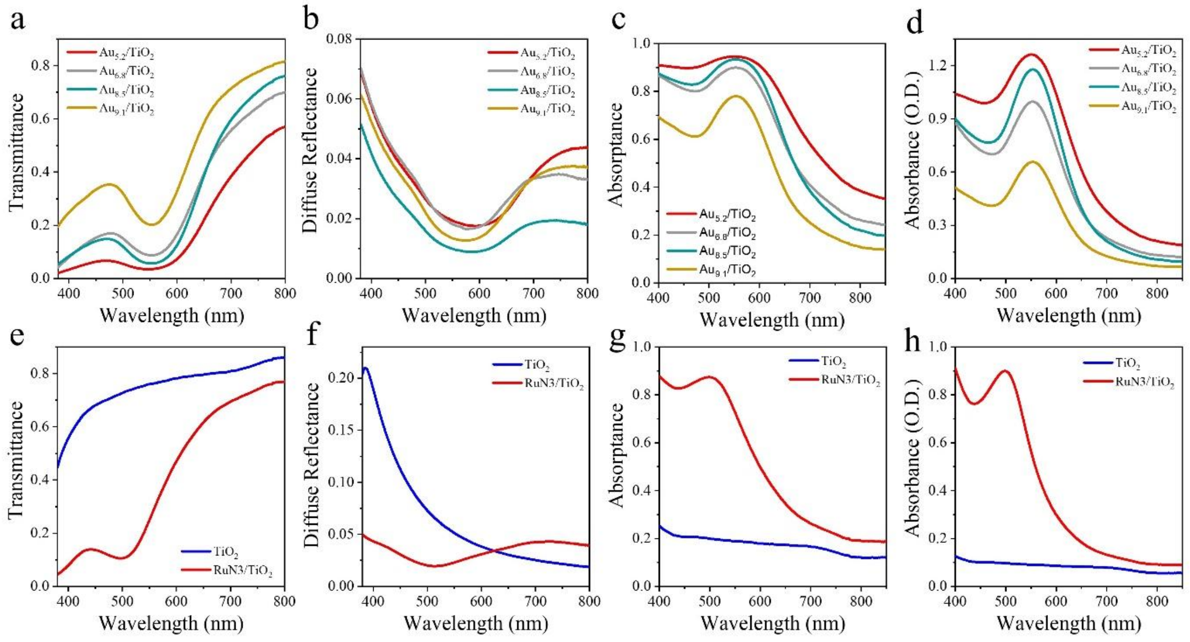

Figure S5: (a, e) Transmission, (b, f) Diffuse reflectance, (c, g) absorptance and (c, g) calculated absorbance spectra of different size Au/TiO_2(a-d), $RuN3/TiO_2$(e-h), and TiO_2 films (e-h).

SI4: Calculation of True Absorbance

Total absorption can be calculated using following equation,

$$A = 1 - T - S$$

Where, A is absorptance, T transmittance, and S is diffuse reflectance/scattering.

Then, we calculated Absorbance in the unit of optical density by the following question,

$$\text{Absorbance (O.D.)} = -log_{10}(1 - absorption\ (A))$$

The absorptance spectra of Au/TiO_2, as depicted in Figure S5c, were derived from the data presented in Figure S5a and S5b.

SI5. Fitting Parameters of Absorption/Extinction Spectra of Colloidal Au NPs and Au/TiO_2 Films

The absorption/extinction profile of the Au NPs is modeled as a composite function comprising of a Lorentzian representation of the surface plasmon resonance (SPR) band, and an interband transition function with an onset energy at approximately 2.4 eV.[3] The Lorentzian function can be written as,

$$f_{SPR} = \frac{A}{\pi} \frac{\frac{w}{2}}{(E-E_c)^2 + \left(\frac{w}{2}\right)^2} \qquad \text{(S1)}$$

Where, A is the amplitude, w is the full width half maxima of the function and E_c is the peak position of the SPR.

The interband absorption, f_{inter} can be expressed as follows,

$$f_{inter}(E) = \int A(E - E_{g'})^B \times \frac{1}{\sqrt{2\pi}\sigma} exp\left[-\frac{(E_{g'} - E_g)^2}{2\pi\sigma^2}\right] dE_{g'} \qquad \text{(S2)}$$

The optical absorption of the material under study involves an interband transition with an onset energy, E_g, which is characterized by the function A(E−E_g). However, due to the presence of minute inhomogeneous particle sizes, E_g becomes uncertain and exhibits a Gaussian distribution with a standard deviation of σ. Consequently, the actual interband transition response, f_{inter}, is a convolution of the interband transition function with this Gaussian distribution of E_g. To obtain the solution, the above equation was numerically integrated over the energy range from 1.6 eV to 3.2 eV. The resulting numerical integration provided the fitting results, which are detailed in Table S2,

and the corresponding plots are depicted in Figure S6 and Figure S7 for Au/TiO_2 films and colloidal Au NPs. This numerical approach allows for a comprehensive analysis of the interband transition behavior in the presence of the inhomogeneous distribution of particle sizes and provides valuable insights into the optical properties of the material. We observed that there are increment in FWHM of LSPR in the presence of TiO_2 films, due to an additional charge transfer state or chemical interface damping (CID) mechanism. The slope of the plasmon linewidth (surface and chemical interface damping) and interface (bulk damping) are summarized in Table S3 from the linear fitting of the plasmon peaks.

In Au/TiO_2 films, discernible red shifts of 0.14 eV (Figure S4b) in the peak positions of the SPR bands, coupled with noticeable broadening of the bands, signify compelling alterations in the electronic environment surrounding the Au NPs upon their integration with TiO_2. These pronounced spectral changes are indicative of robust electronic coupling between the Au and TiO_2 domains, underscoring the presence of substantial interactions between the materials in the composite system. As such, the evidence strongly supports the notion of profound and influential interactions between the Au and TiO_2 domains in the Au/TiO_2 films, which significantly impact their optical properties.

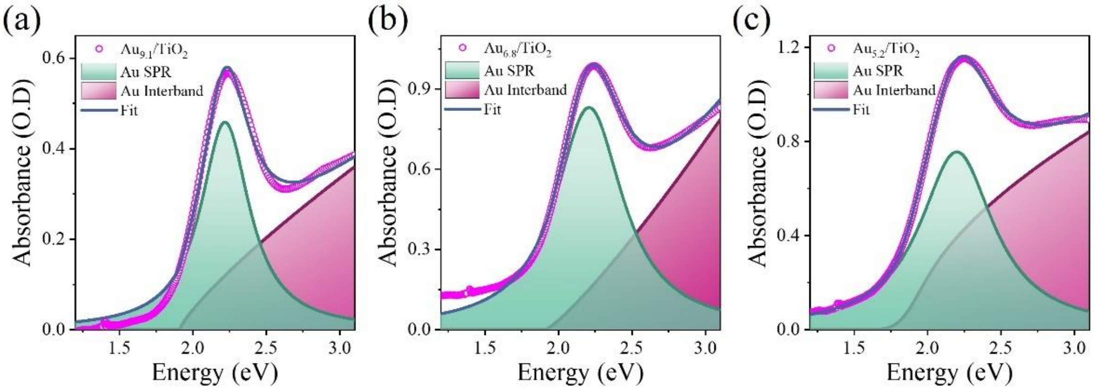

Figure S6: UV-Vis absorption spectra of Au/TiO_2 heterostructures with different Au sizes (a) 9.13 ± 1.43 nm (b) 6.86 ± 1.03 nm and (c) 5.25 ± 0.89 nm. The pink open circles denote the experimental data. The dark cyan, dark red lines and bluegray color represent the Au SPR band, interband transition and accumulative fitting line, respectively.

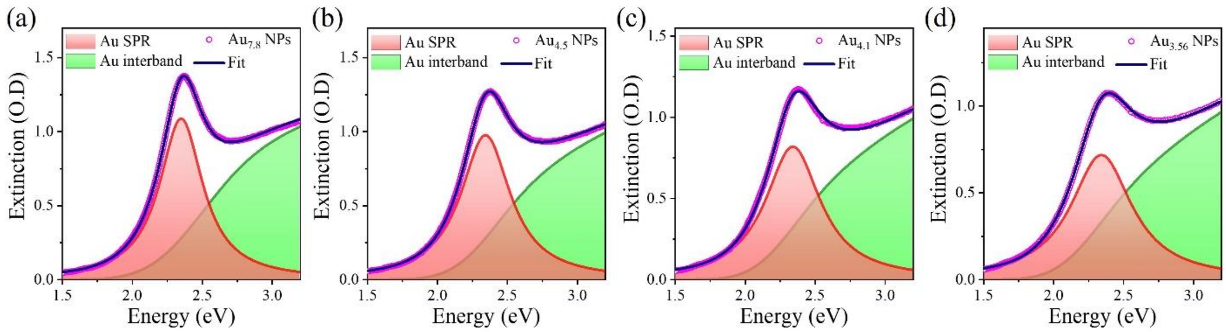

Figure S7: UV-Vis extinction spectra of different Au NPs size (a) 7.86 ± 0.65 nm (b) 4.56 ± 0.29 nm, (b) 4.1 ± 1.0 nm and (d) 3.56 ± 1.29 nm. The pink open circles are experimental data. The red, green lines and dark blue color represent Au SPR band, interband transition and accumulative fitting line, respectively.

Table S2: Fitting parameters of SPR band of Au NPs and Au/TiO_2 films.

Size (nm)	Au NPs				Au/TiO_2 films			
	7.86± 0.65	4.56± 0.29	4.1± 1.00	3.56± 1.29	9.13 ± 1.43	8.51 ± 1.49	6.86 ± 1.03	5.25 ± 0.89
SPR width (eV)	0.378± 0.014	0.432± 0.010	0.479± 0.006	0.533± 0.004	0.404± 0.015	0.444 ± 0.010	0.536 ± 0.006	0.610 ± 0.006

Table S3: Fitting components of SPR vs (1/R) of Au and Au/TiO_2 NPs.

System	Intercept (a)	Slope (b)
Au NPs	0.21 ± 0.06	0.57 ± 0.11
Au/TiO_2 films	0.21 ± 0.06	1.10 ± 0.18

SI6: Plasmon Damping Fitting

Assuming negligible inhomogeneous broadening due to size and shape distributions, the total plasmon damping rate can be related to the SPR width ($\gamma_{tot} = \frac{\pi \Delta E_{obs}}{h}, in\ s^{-1}$), where ΔE_{obs} is observed total SPR bandwidth,.[1,2,4] The total damping rates are the sum of bulk (γ_{bulk}), surface (γ_{surf}) and interfacial or CID (γ_{int}) damping rates , as shown in Eq. (S1).[5,6,7]

$$\gamma_{tot} = \gamma_{bulk} + \gamma_{surf} + \gamma_{int} \quad \text{(S3)}$$

It has been shown that the surface dephasing rates (γ_{surf}) becomes significant when the size of NPs are below the characteristic electron mean free path within the system (~50 nm for Au) and is linearly dependent on 1/R according to $\gamma_{surf} = A\frac{v_F}{R}$, in which R is the average radius of Au particles.[8, 9] The interfacial or CID term is caused by the interaction of Au and TiO_2,[7, 10, 11] and has been shown to follow $\gamma_{int} = B\frac{v_F}{R}$.[10, 11, 12] Here, A and B are scaling factors and v_F is the Fermi velocity of an electron in Au (~1.4×10^6 $m.s^{-1}$).[13] We have fitted SPR width vs (1/R) data following our previous investigation,[2] and extracted results reveal that $\gamma_{bulk} = 0.5 \times 10^{14}\frac{1}{s}\,(or\ 0.21\ eV)$, and the scaling factor A, and B is 0.41 ± 0.08, and 0.38 ± 0.12 eV•fs, consistent with previously reported results (Table S3).[2, 4] The results confirm that CID by TiO_2 plays an important role in the damping of Au plasmon band for Au/TiO_2.

SI7. Mid-IR Transient Absorption Setup

A Coherent Systems Astrella Ti:Sapphire regenerative amplifier system laser system was utilized, operating at a central wavelength of 800 nm with a power output of 5 W and an ultrashort pulse width of 35 fs. The laser system operated at a repetition rate of 1 kHz. This system was coupled with both infrared (IR) and visible optical parametric amplifiers (OPAs) to enable transient infrared experiments. The pump beams employed for the experiments were generated from the visible OPA through the processes of sum frequency generation (SFG) and second harmonic generation (SHG) using a Beta Barium Borate (BBO) crystal for wavelength conversion. Pump pulses were chopped with an electrical chopper with a frequency of 500 Hz. The mid-IR probe was generated by another OPA (OperA Solo, Coherent) via difference frequency generation by the mixing signal and idler in a AgGaAs DFG crystal. Horiba iHR 320 monochromators with a 50 grooves/mm grating and nitrogen cooled 128×128-pixel MCT detector (Teledyne, PhaseTech) were utilized to measure the probe intensity with and without pump laser pulse. Transient absorption was acquired using PhaseTech's software, QuickControl.

SI8: Background correction and QEs of the hot electron transfer in a scattering sample

Here the system (Au/TiO_2, or $RuN3/TiO_2$) under experimental conditions consists of two species Au and TiO_2. Consequently, both species can absorb photon light, and can contribute to observed TAIR probe signal. Before estimating QEs of the Au/TiO_2 system, it needs to correct the scattering light and subtract the TiO_2 absorption. However, the absorption of TiO_2 is large in bare TiO_2 as compared to the Au/TiO_2 film due to the presence of the Au NPs.

The absorption due to TiO_2 in TiO_2 films can be expressed as,

$$I'_{TiO2} = I_0(1 - R' - T') \quad \text{(S4)}$$

Whereas I_0 is the initial intensity, R' is the diffuse reflectance, and T' is the transmittance of the bare TiO_2 film.

Now, the absorption of TiO_2 in Au/TiO_2 films,

$$I_{TiO2} = I_0(1 - R - T)\frac{OD_{TiO2}}{OD_{Au}+OD_{TiO2}} \quad \text{(S5)}$$

Similarly, the absorption of Au NPs in the Au/TiO_2 films,

$$I_{Au} = I_0(1 - R - T)\frac{OD_{Au}}{OD_{Au}+OD_{TiO2}} \quad \text{(S6)}$$

Here, R is the diffuse reflectance, and R is the transmittance of the bare Au/TiO_2 film.

Therefore, the signal intensity of bare TiO_2 films is,

$$S'_{TiO2} = C_1 I_0(1 - R' - T') \quad \text{(S7)}$$

So, the proportionality constant, which is related to absorption cross section can be expressed as,

$$C_1 = \frac{1}{I_0(1-R'-T')} S'_{TiO2} \quad \text{(S8)}$$

Signal intensity of TiO_2 and Au NPs in Au/TiO_2 films,

$$S_{TiO2} = C_1 I_{TiO2} = C_1 I_0(1 - R - T)\frac{OD_{TiO2}}{OD_{Au}+OD_{TiO2}} \quad \text{(S9)}$$

$$S_{Au} = C_2 I_{Au} = C_2 I_0(1 - R - T)\frac{OD_{Au}}{OD_{Au}+OD_{TiO2}} \quad \text{(S10)}$$

The final expression for the TiO_2 signal in the Au/TiO_2 films,

$$S_{TiO2} = \frac{(1-R-T)\frac{OD_{TiO2}}{OD_{Au}+OD_{TiO2}}}{(1-R'-T')} S'_{TiO2} \quad \text{(S11)}$$

The total corrected injected electron in Au/TiO_2 films can be expressed as,

$$S_{Au} = S_{Au/TiO2} - S_{TiO2} = S_{Au/TiO2} - \frac{(1-R-T)\frac{OD_{TiO2}}{OD_{Au}+OD_{TiO2}}}{(1-R'-T')} S'_{TiO2} \quad \text{(S12)}$$

Similarly, corrected electron injection signal for the RuN3/TiO_2 films,

$$S_{RuN3} = S_{RuN3/TiO2} - S_{TiO2} = S_{RuN3/TiO2} - \frac{(1-R-T)\frac{OD_{TiO2}}{OD_{RuN3}+OD_{TiO2}}}{(1-R'-T')} S'_{TiO2} \quad \text{(S13)}$$

Quantum efficiency of electron injection in Au/TiO_2 film,

$$\phi(Au) = M\frac{S_{Au}}{I_{Au}} \quad \text{(S14)}$$

Quantum efficiency of electron injection in RuN3/TiO_2 film,

$$\phi(RuN3) = M\frac{S_{RuN3}}{I_{RuN3}} \quad \text{(S15)}$$

Final expression for determining Quantum efficiency of electron injection in Au/TiO_2 (using RuN3/TiO_2 as a known reference) is:

$$\phi(Au) = \left(\frac{\left(\frac{S_{Au}}{I_{Au}}\right)}{\left(\frac{S_{RuN3}}{I_{RuN3}}\right)}\right)\phi(RuN3) \quad \text{(S16)}$$

Here, we assume 100% electron transfer efficiency in RuN3/TiO_2 and compared with Au/TiO_2 films.

SI9. Electron Injection Kinetics of Au/TiO_2 and RuN_3/TiO_2 Films

The electron injection kinetics for the RuN3/TiO_2 and Au/TiO_2 systems can be fit by Equations (S17) and (S18), respectively, following previous reports.[4] Notably, in the case of RuN3/TiO_2, the electron absorption signal results from the convolution of the evolving electron population ($N_e(t)$) and the time-varying absorption cross-section ($\sigma_h(t)$), as described by Equation (S17).

$$S_{RuN3}(t) = IRF \otimes \{A_0\left[1-\exp\left(-\frac{t}{t_0}\right)\right] \times \sigma_h(t) + A_1\left[1-\exp\left(-\frac{t}{t_1}\right)\right] + A_2[1-\exp(-\frac{t}{t_2})]\} \quad \text{(S17)}$$

Here, A_0 and τ_0 are the amplitude and time constant of the fast component of electron injection to TiO_2 from the initially excited RuN3 state, whereas, A_1, A_2, τ_1 and τ_2 are the amplitude and time constant of the slow components of electron population injected from the relaxed excited state to near the band edge. The cross-section of electron absorption can be described as,

$$\sigma_h(t) = [0.4 \times \exp\left(-\frac{t}{90}\right) + 0.6 \times \exp\left(-\frac{t}{50000}\right)] \quad \text{(S18)}$$

The instrument response function (IRF) was determined via an experimental procedure entailing the measurement of the electron intraband absorption within a silicon wafer, conducted under the same experimental conditions. The observed rise kinetics of the intraband signal can be well modeled by a Gaussian instrument response function with a full-width half-maximum (FWHM) of 160 femtoseconds for excitations at wavelengths of 500 nm. In Eq. (S17), we have assumed negligible back electron transfer on the <1ns time scale.

The data set was fit by Equation (S17), leading to the determination of the maximum population of injected electrons within the RuN3/TiO_2 material in Table S4, Table S5, and Table S6. This peak population was identified at approximately ~300 picoseconds, at which delay time, the injected hot electrons have already relaxed to the edge of the conduction band (CB), and the charge recombination process has not yet commenced.

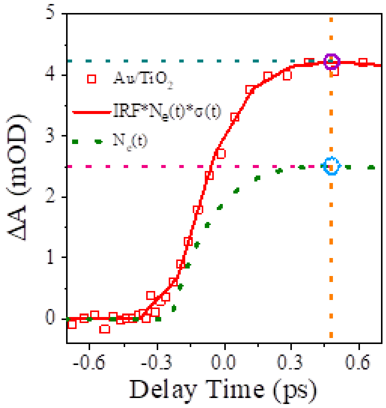

Figure S8: The squares are the experimental results, and the red solid line is the fitted kinetics, which is the convolution of IRF, electron population and absorption cross-section. The dashed green line is the electron population.

The transient IR kinetics of Au/TiO_2 are fit to Eq.(19), in which we assume that all electrons are injected above the conduction band edge at the ultrafast time scale due to the fast electron

relaxation wihin Au and there is a multiple exponential back electron transfer processes on the < 1 ns time scale.

$$S_{Au/TiO_2(t)} = IRF \otimes \{[-(A_1 + A_2 + A_3)\exp\left(-\frac{t}{t_f}\right) + A_1 \exp\left(-\frac{t}{t_1}\right) + A_2 \exp\left(-\frac{t}{t_2}\right) + A_3 \exp(-\frac{t}{t_3})] \times \sigma_h(t)\}$$

(S19)

In Figure S8, the green short-dashed line represents the injected electron population from Au to TiO_2 over time. The amplitude of the injected electron signal in Au/TiO_2 can be determined by the following two steps: 1. Identifying the maximum value from the fitted kinetics (solid red line); 2. Multiplying this maximum value by a coefficient of 0.6.

SI10. Fowler Model for PHET QEs Calculation

Figure S9 illustrates the size-dependent electron injection kinetics into the TiO_2 conduction band upon 400, and 600 nm excitation.

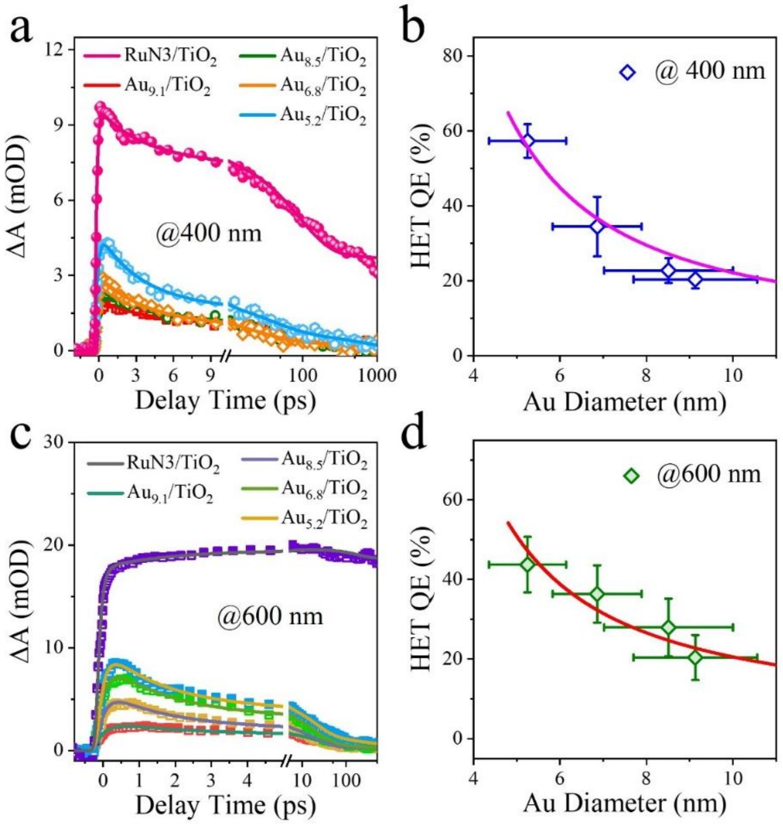

Figure S9: (a,c) Size dependent electron injection kinetics and (b,d) hot electron transfer quantum efficiency in Au/TiO_2 measured with 400 nm (a,b) and 600 nm (c,d) excitation. The excitation fluence are 0.41 W/cm^2 at 400 nm and ~0.91 W/cm^2 at 600 nm, respectively.

Table S4: Fitting parameters of electron injection kinetics of Au/TiO_2 films (of varying Au NP size) under 400 nm excitation (0.41 W·cm^{-2}).

Au Size (nm)	τ_f (ps)	A_1	τ_1 (ps)	A_2	τ_2 (ps)	A_3	τ_3 (ps)	Half Life (ps)
9.13±1.43	0.20 ± 0.03	41%	3.4 ± 0.9	26%	35 ± 15	33%	>1ns	10.2
8.51±1.49		38%		38%		24%		
6.86±1.03		46%		34%		20%		
5.25±0.89		58%		20%		22%		

Table S5: Fitting parameters of electron injection kinetics of Au/TiO_2 films (of varying Au NPs size) under 500 nm excitation (0.61 W·cm^{-2}).

Au Size (nm)	τ_f (ps)	A_1	τ_1 (ps)	A_2	τ_2 (ps)	A_3	τ_3 (ps)	Half Life (ps)
9.13±1.43	0.17 ± 0.03	43%	2.4 ± 0.7	26%	17.6 ± 18	31%	>1ns	6.6
8.51±1.49		33%		36%		30%		
6.86±1.03		57%		15%		28%		
5.25±0.89		60%		16%		24%		

Table S6: Fitting parameters of electron injection kinetics of Au/TiO_2 films (of varying Au NPs size) under 600 nm excitation (0.91 W·cm^{-2}).

Au Size (nm)	τ_f (ps)	A_1	τ_1 (ps)	A_2	τ_2 (ps)	A_3	τ_3 (ps)	Half Life (ps)
9.13±1.43	0.17 ± 0.03	55%	1.53 ± 0.34	38%	21.3 ±5.3	7%	>1ns	2.7
8.51±1.49		56%		35%		9%		
6.86±1.03		52%		18%		30%		
5.25±0.89		65%		18%		17%		

Table 7: QEs of different size Au NPs embedded in TiO_2 films under 400, 500, and 600 nm respectively.

Au NPs Size	400 nm	500 nm	600 nm
5.25±0.89	57.32 ± 4.47	56.23 ± 11.24	43.72 ± 5.63
6.86±1.03	34.46 ± 7.95	34.78 ± 10.93	36.36 ± 7.24
8.51±1.49	22.75 ± 3.29	29.11 ± 2.95	27.96 ± 7.20
9.13±1.43	20.36 ± 2.40	18.32 ± 1.86	20.38 ± 6.95

SI11. Pump Power Dependent TA Kinetics of Au/TiO_2 and RuN_3/TiO_2 Films

Pump power dependent transient absorption (TA) kinetics of Au/TiO_2 and RuN3/TiO_2 films were performed. We have presented TAIR of RuN3/TiO_2 in Figure S10. The TA kinetics exhibit a notable characteristic: the absence of pump power dependence. This observation suggests that the kinetics remain unaffected by variations in the pump power employed during the experimental procedure. Such independence underscores the robustness and stability of the TA kinetics, indicating consistent behavior across different pump intensities. Hot electron population kinetics from Au NPs are demonstrated in Figure S11.

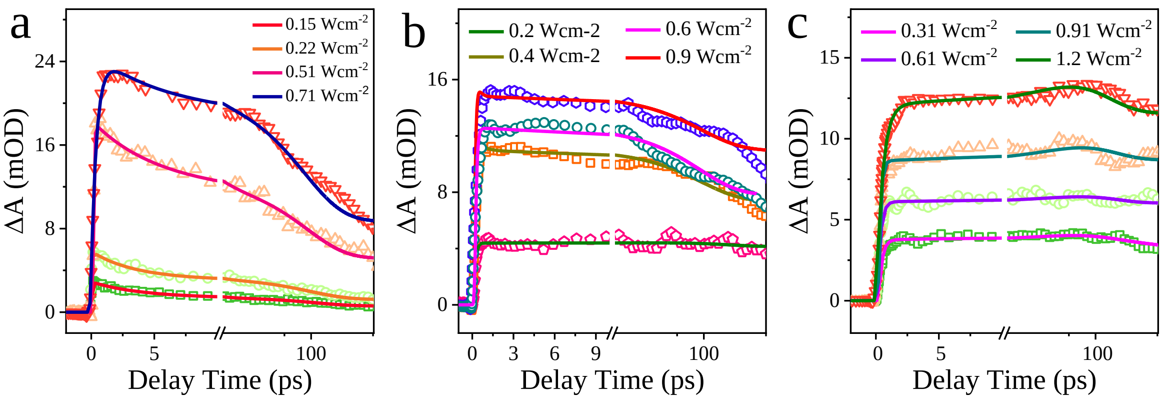

Figure S10: Hot electron injection kinetics in RuN3/TiO_2 films under different pump fluence. (a) 400 nm, (b) 500 nm, and (c) 600 nm, respectively. The scattered open points are the experimental data and solid lines are the fits, respectively.

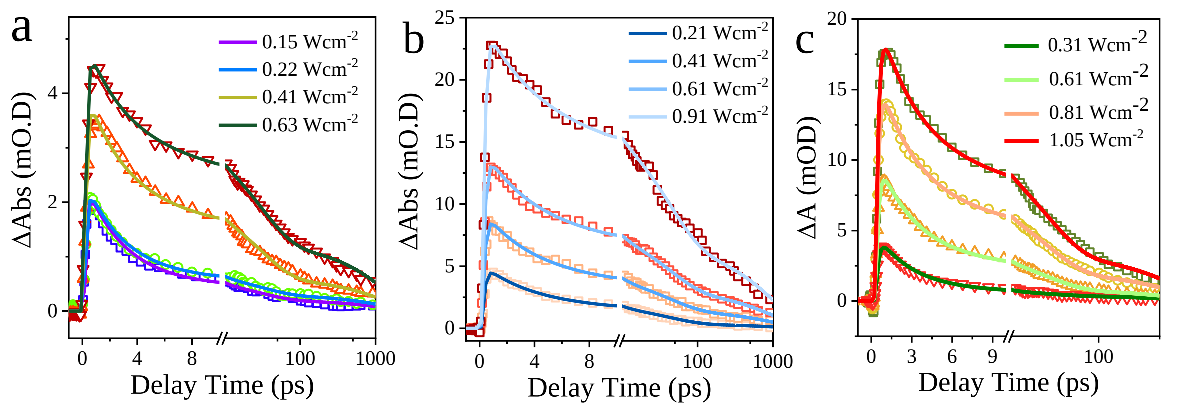

Figure S11: Hot electron injection kinetics in $Au_{8.5}/TiO_2$ films under different pump fluence. (a) 400 nm, (b) 500 nm, and (c) 600 nm, respectively. The scattered open points are the experimental data and solid lines are the fits, respectively.

Figure S12 presents the size-dependent electron injection amplitude from Au NPs for pump excitations at 400 nm, 500 nm, and 600 nm. The injected electron mid-IR intraband absorption amplitude can be well fitted by a linear dependence on the excitation power, which implies negligible contributions of the two-photon excitation process.

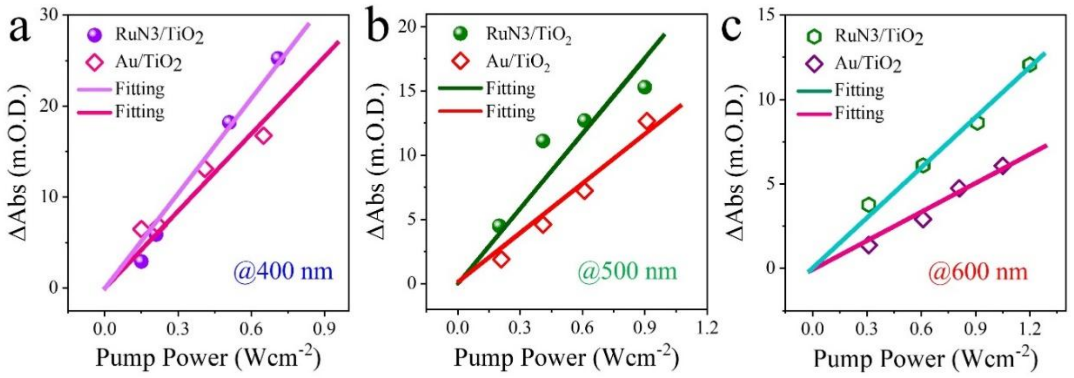

Figure S12: Linear fitting of hot electron injection amplitude of Au/TiO_2 and $RuN3/TiO_2$ films under (a) 400 nm, (b) 500 nm, and (c) 600 nm pump laser excitation, respectively.

SI12. Control Experiment and Electron Injection Signal of Au/Al_2O_3 and Bare TiO_2 Films

Figure S13 represents TAIR electron injection kinetics of bare sapphire (Al_2O_3), TiO_2 and $Au_{8.5}/TiO_2$ films. To ascertain that the observed electron signal in the $Au_{8.5}/TiO_2$ sample originates from electron injection from Au to TiO_2, rather than from Au itself, comparative analysis was conducted with an $Au_{8.5}/Al_2O_3$ film. This shows that most of the signal amplitude comes from the Au electron injection process. We have observed a small TAIR signal for bare TiO_2 film for all the pump excitation (400, 500, and 600 nm), due to the presence of mid band gap defect state in TiO2 films. The presence of defect state is also confirmed by steady state DRS results of TiO_2 film (Figure S5h).

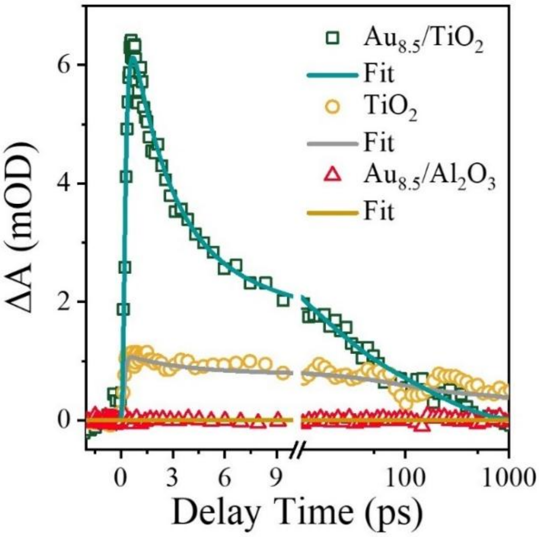

Figure S13: Transient kinetics of TiO_2, TiO_2, and $Au_{8.5}/Al_2O_3$ films upon 500 nm pump excitation.

REFERENCES

(1) Peng, S.; Lee, Y.; Wang, C.; Yin, H.; Dai, S.; Sun, S. A Facile Synthesis of Monodisperse Au Nanoparticles and Their Catalysis of CO Oxidation. *Nano Res* **2008**, *1* (3), 229–234.

(2) Song, J.; Long, J.; Liu, Y.; Xu, Z.; Ge, A.; Piercy, B. D.; Cullen, D. A.; Ivanov, I. N.; McBride, J. R.; Losego, M. D.; Lian, T. Highly Efficient Plasmon Induced Hot-Electron Transfer at Ag/TiO2Interface. *ACS Photonics* **2021**, *8* (5), 1497–1504.

(3) Liu, Y.; Chen, Q.; Chen, Q.; Cullen, D. A.; Xie, Z.; Lian, T. Efficient Hot Electron Transfer from Small Au Nanoparticles. *Nano Lett* **2020**, *20* (6), 4322–4329.

(4) Tian, Y.; Tatsuma, T. Mechanisms and Applications of Plasmon-Induced Charge Separation at TiO_2 Films Loaded with Gold Nanoparticles. *J. Am. Chem. Soc.* **2005**, *127* (20), 7632-7637.

(5) Kittel, C.; McEuen, P. *Introduction to solid state physics*; John Wiley & Sons, 2018.

(6) Besteiro, L. V.; Kong, X.-T.; Wang, Z.; Hartland, G.; Govorov, A. O. Understanding Hot-Electron Generation and Plasmon Relaxation in Metal Nanocrystals: Quantum and Classical Mechanisms. *ACS Photonics* **2017**, *4* (11), 2759-2781.

(7) Zhang, W.; He, Y.; Zhang, M.; Yin, Z.; Chen, Q. Raman scattering study on anatase TiO2 nanocrystals. *Journal of Physics D: Applied Physics* **2000**, *33* (8), 912.

(8) Hövel, H.; Fritz, S.; Hilger, A.; Kreibig, U.; Vollmer, M. Width of cluster plasmon resonances: Bulk dielectric functions and chemical interface damping. *Physical Review B* **1993**, *48* (24), 18178.

(9) Zhang, Z.; Zhang, C.; Zheng, H.; Xu, H. Plasmon-Driven Catalysis on Molecules and Nanomaterials. *Accounts of Chemical Research* **2019**, *52* (9), 2506-2515.

(10) Foerster, B.; Joplin, A.; Kaefer, K.; Celiksoy, S.; Link, S.; Sönnichsen, C. Chemical Interface Damping Depends on Electrons Reaching the Surface. *ACS Nano* **2017**, *11* (3), 2886-2893.

(11) Li, J.; Cushing, S. K.; Meng, F.; Senty, T. R.; Bristow, A. D.; Wu, N. Plasmon-induced resonance energy transfer for solar energy conversion. *Nature Photonics* **2015**, *9* (9), 601-607.

(12) Govorov, A. O.; Lee, J.; Kotov, N. A. Theory of plasmon-enhanced Förster energy transfer in optically excited semiconductor and metal nanoparticles. *Physical Review B* **2007**, *76* (12), 125308-125308.

(13) Kreibig, U.; Vollmer, M. *Optical properties of metal clusters*; Springer Science & Business Media, 2013.
